# Assessment of Oral Microbial Viability by 2,6-Dichlorophenolindophenol a Redox Agent

**DOI:** 10.3390/antibiotics14060590

**Published:** 2025-06-07

**Authors:** Prem K. Sreenivasan, Violet I. Haraszthy

**Affiliations:** 1 HITLAB, 3960 Broadway, New York, NY 10032, USA; 2 Adjunct Faculty, JSS Academy of Higher Education and Research, Mysuru 570 015, India; 3 Department of Restorative Dentistry, University at Buffalo, Buffalo, NY 14214, USA; vh1@buffalo.edu

**Keywords:** antibiotics, bacteria, cetylpyridinium chloride, chlorhexidine, culture, DCIP, mouthrinse, redox, toothpaste, triclosan, viability

## Abstract

**Background/Objectives:** This investigation evaluated 2,6-Dichlorophenolindophenol (DCIP), a redox dye, as a colorimetric reagent for rapid quantification of oral bacteria and examined the antimicrobial effects of oral hygiene formulations. **Methods/Results:** Viable microbial cells reduce DCIP, resulting in a loss of its blue color which can be measured spectrophotometrically. Strains of *Actinomyces viscosus*, *Veillonella atypica*, *Aggregatibacter actinomycetemcomitans*, *Streptococcus mutans* and *Candida albicans* grown in the laboratory reduced DCIP. Significant correlations between increasing viable plate counts and DCIP reduction were noted for strains of oral organisms. Intact microbial cells reduced DCIP, with insignificant reductions observed by spent microbial media or bacteria free culture media. Organisms inactivated by either heat or cold demonstrated significantly lower DCIP reduction in comparison to metabolically intact organisms grown under optimal conditions. **Conclusions:** DCIP reduction provided a rapid and accurate method to evaluate antimicrobial effects of clinical proven mouthwashes formulated with cetylpyridinium chloride or chlorhexidine and toothpastes for a range of oral bacteria. Together, these results identify a rapid, low-cost method using common laboratory equipment to enumerate oral organisms with a visual outcome.

## 1. Introduction

Epidemiological surveys reveal that oral diseases such as dental caries, gingivitis and periodontal diseases are widespread globally [1]. Current practices in clinical dentistry highlight the importance of effective control of the dental plaque and the microorganisms found in the human mouth [2]. Self-care, reflected by effective daily dental hygiene practices, remains an important component to maintain oral hygiene [3]. However, despite many efforts, including in education, research and other public health programs, a majority of individuals fail to maintain the required levels of daily oral hygiene [2,3].

The inclusion of antimicrobials in daily use oral hygiene formulations is a widely utilized strategy to help improve oral hygiene [3,4]. Available in the literature are many clinical studies evaluating a variety of formulations with antimicrobial agents. The daily use of mouthrinses and dentifrices formulated with several well-known antimicrobial ingredients demonstrates reductions in dental plaque and gingivitis in clinical studies [3,4].

The development and optimization of effective oral hygiene formulations with antimicrobial agents [5,6] requires an extensive array of efforts with the quantification of microbial viability serving as an important measure of formulation efficacy. Traditional microbiological techniques such as plating bacteria on agar, while still in use, are increasingly being replaced by faster and less labor-intensive biochemical methods. Some of these are based on changes in color or fluorescence of resazurin- or tetrazolium-derived redox reagents upon their reduction by metabolically active organisms. While these reagents have been predominantly developed for measurement of cytotoxicity of mammalian cells, many of them have been shown to work well with bacteria [7,8,9,10].

2,6-Dichlorophenolindophenol (DCIP) is another redox dye amenable to calorimetrically detect reduction by living cells [10]. This is due to the fact that DCIP is able to act as a substitute for NADP^+^, which is reduced by the cell to make NADPH. Thus, due to this substitution, cellular viability can be monitored calorimetrically using standard laboratory equipment. In its oxidized form DCIP is blue in color, and colorless when reduced. If the cell is viable, one would anticipate the reduction in DCIP and a reduction in the color intensity vs. dead cells, which would not reduce DCIP and maintain the blue color of the oxidized DCIP.

DCIP has been employed in metabolic and cytotoxicity assays for bacteria and mammalian cells [11,12,13,14,15], and yeast cells [16], where its reduction was detected both spectrophotometrically and electrochemically [11,12,13,14,15,16]. The present study evaluated the utility of DCIP reduction as a simple method to enumerate oral bacteria and correlate these with microbial culture. These methods were used to evaluate the cytotoxic effect of oral hygiene products and antibiotics.

## 2. Results

### 2.1. Evaluation of DCIP Reduction in Quantification of Viable Microbial Cells

To evaluate the utility of DCIP reduction as a rapid method of measuring numbers of viable microorganisms, experiments were designed to compare it to the agar plating method of microbial quantification. Bacterial cells of indicated strains were serially diluted and quantified by both methods in parallel.

**Figure 1:**
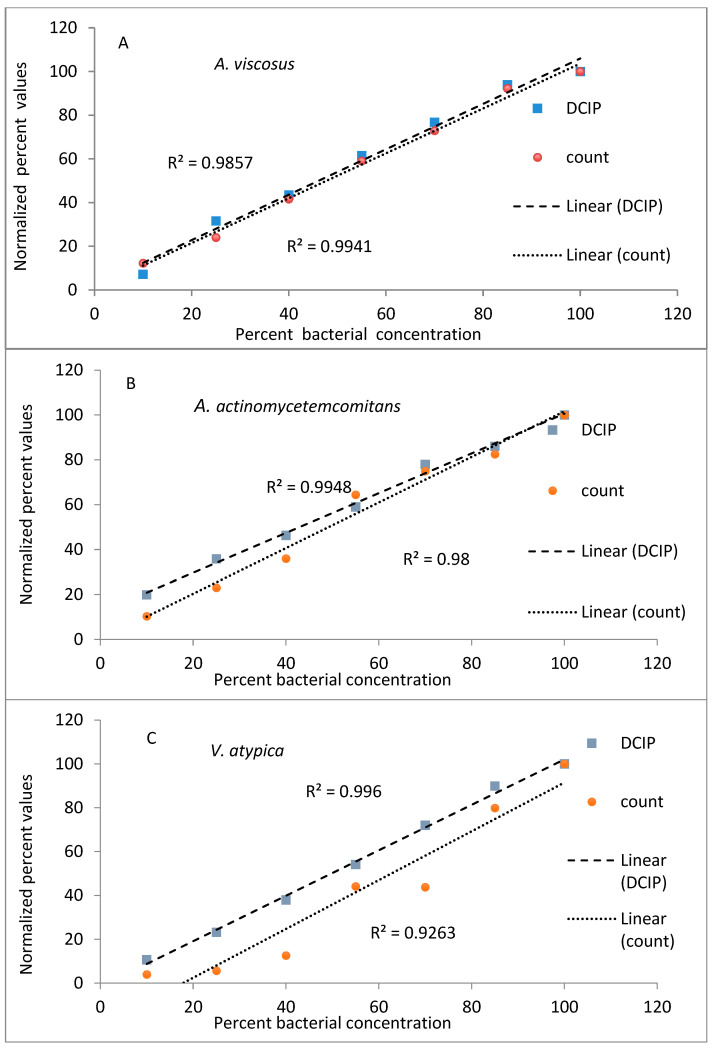

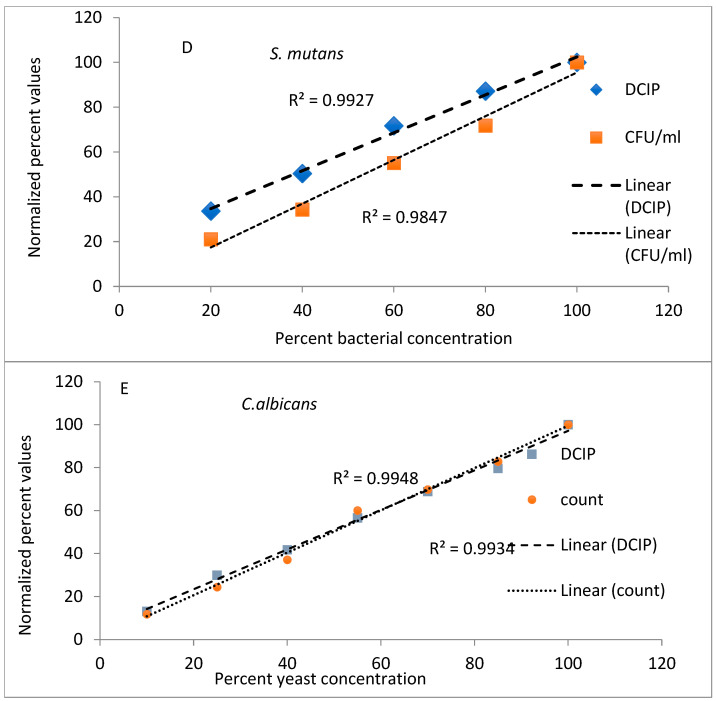
Relationship between viable cell counts and DCIP-reducing activities of oral organisms *A. viscosus* (**A**), *A. actinomycetemcomitans* (**B**) *V. atypica* (**C**), *S. mutans* (**D**) and *C. albicans* (**E**). For each organism, analyses indicate statistically significant correlations between DCIP results and corresponding viable counts (*p* < 0.05).

The results from evaluations (reduction of DCIP and bacterial counts, expressed as a percent of values obtained for undiluted bacteria) plotted against known dilutions of bacteria are shown in Figure 1. In the experiments performed on *A. viscosus* and *A. actinomycetemcomitans* both methods provided comparable results, with coefficients of determination greater than 0.98 for both methods and species (Figure 1A,B). Similar results were obtained to those obtained for other oral organisms *V. atypica, S. mutans* and *C. albicans* (Figure 1C,E) representing significant results (*p* < 0.05).

### 2.2. Location of DCIP-Reducing Activity in Microbial Culture

DCIP-reducing activities were measured in an unfractionated suspension of *A. viscosus* bacteria as well as in pellets and in supernatants following centrifugation. Almost all of the activity in bacteria fractionated by centrifugation was concentrated in pellet (80 ± 0.93) with values comparable to the unfractionated bacteria (79 ± 1.78), indicating that it was associated with the bacterial cells. This is consistent with earlier reports pointing to intracellular NADH and NADPH as electron donors in reduction of DCIP and other redox dyes [10,17]. Correspondingly, spent bacterial medium or sterile bacteriological medium devoid of any microorganisms did not reduce DCIP (0 ± 3.02 and 3 ± 1.11, respectively).

### 2.3. The Effect of Temperature on DCIP-Reducing Activity

Bacteria were incubated on ice for up to three hours before brief equilibration to the room temperature followed by measurement of DCIP-reducing activity. Different bacterial species were affected by cold to a different degree, with the activity only slightly diminished for *A. viscosus* (Table 1), while it was lowered by as much as 50% for *V. atypica* and *A. actinomycetemcomitans*.

**Figure 2:**
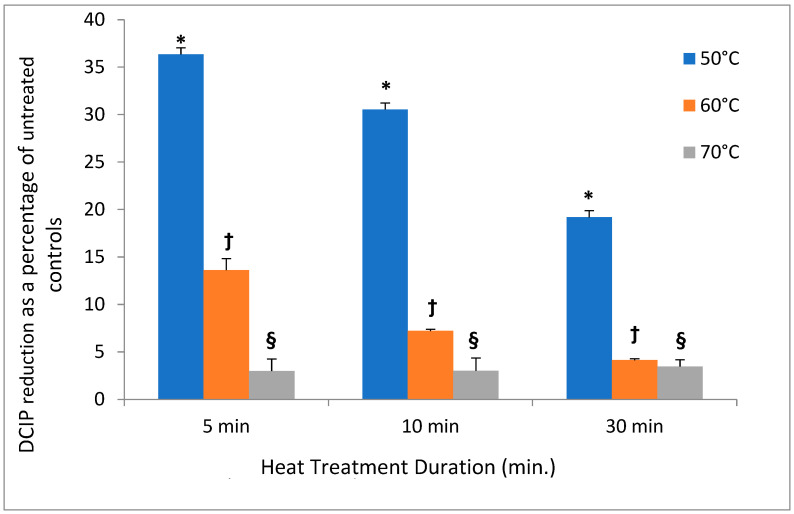
Effect of heat on DCIP reduction by cultures of *A. viscosus.* Bacterial cultures were incubated at the indicated temperature for the specified periods of time prior to the assays. Results are shown as means of triplicate samples ± standard deviation. In comparison to untreated controls, heat treatment of bacterial cultures at 50 °C, 60 °C or 70 °C demonstrated statistically significant reductions in DCIP results by *t*-tests (*p* < 0.05). Symbol * identifies statistically significant differences for the evaluation from all other evaluations. Symbol † identifies statistically significant differences between the 60 °C treatments and from the 50 °C treatment. Symbol § identifies statistically significant differences between the 70 °C treatments and the 50 °C and 60 °C treatments irrespective of durations. There were no differences at 70 °C for any of the treatment durations.

**Figure 3:**
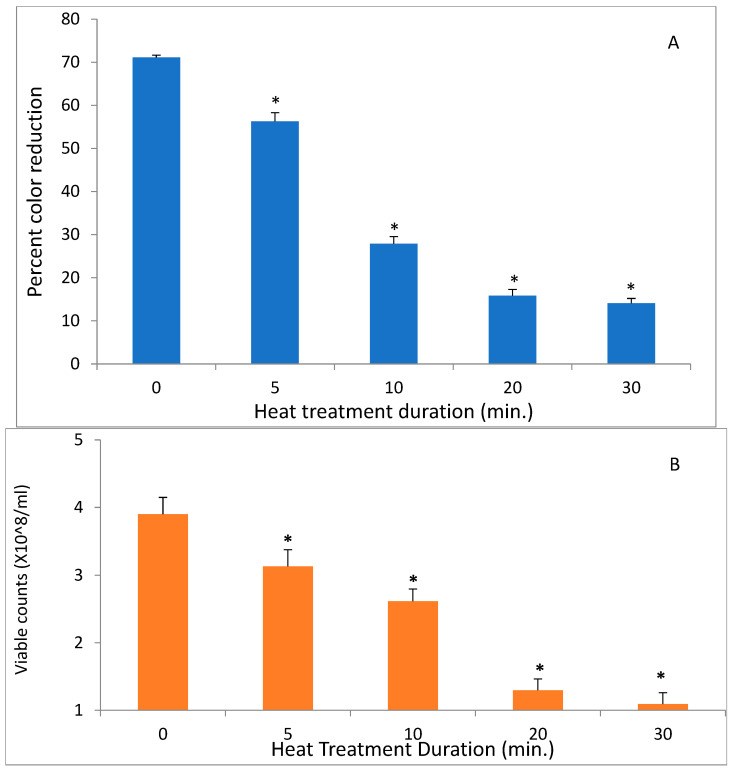
The effect of heat on DCIP-reducing activity (**A**) and on viable cell counts (**B**) of *S. mutans*. Bacterial cultures were incubated at 50 °C for indicated period of time prior to the assays. Results are shown as means of triplicate samples ± standard deviation. Symbol * identifies statistically significant reductions in DCIP results by *t*-tests (*p* < 0.05) after heat treatment of bacterial cultures.

**Figure 4:**
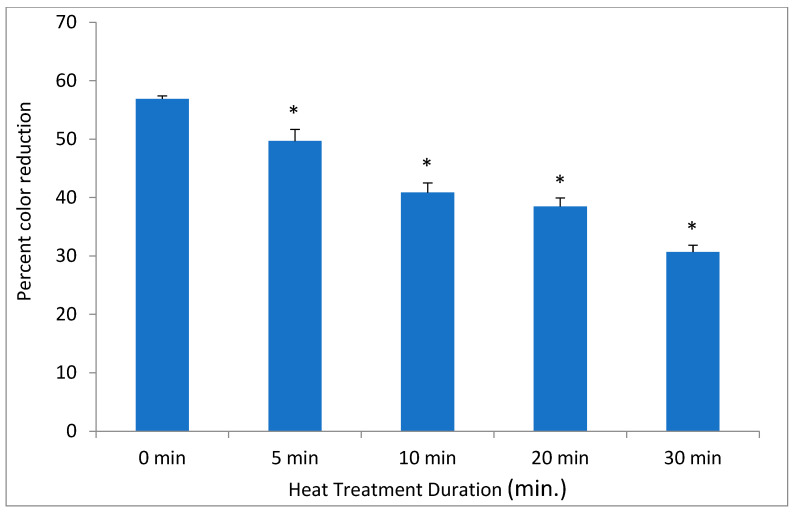
The effect of heat on DCIP-reducing activity on V. atypica. Bacterial cultures were incubated at 50 °C for indicated periods of time prior to the assays. Results are shown as means of triplicate samples ± standard deviation. Symbol * identifies statistically significant reductions in DCIP results by *t*-tests (*p* < 0.05) after heat treatment of bacterial cultures.

In contrast to low temperatures, heat had a much stronger effect on the DCIP-reducing activity of bacteria. Its magnitude depended on both length of incubation and on temperature to which bacteria were exposed (*p* < 0.05). While the reducing activity of *A. viscosus* gradually diminished in the course of 30 min. incubation at 50 °C, higher temperatures led to more abrupt loss of activity (Figure 2). In addition, the investigation evaluated the effect of incubating *S. mutans* at 50 °C on both DCIP-reducing activity and on viability measured by plating on agar. Reduction in microbial viability strongly correlated with loss of reducing activity as demonstrated by an example of *S. mutans* shown in Figure 3 (*p* < 0.05). Shown in Figure 4 is the effect of heat treatment on *V. atypica*.

### 2.4. Evaluation of Oral Care Formulations

[A] Mouthrinses

**Figure 5:**
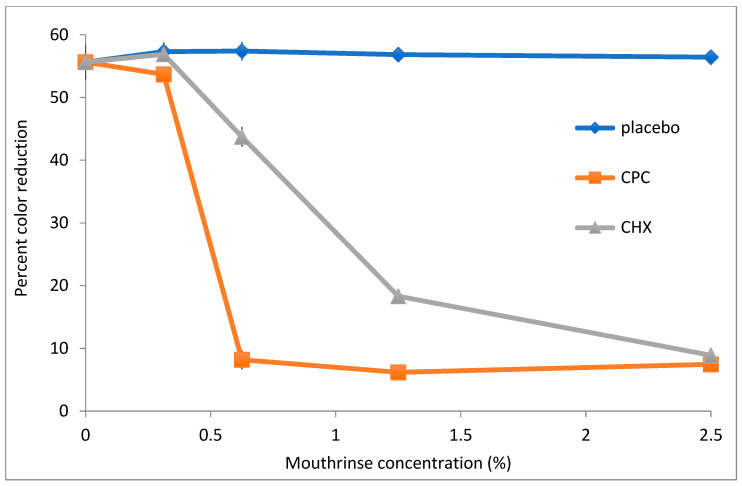
The effect of mouthrinses on DCIP-reducing activity by *A. viscosus.* Bacteria were incubated for 20 min with indicated concentrations of mouthwashes formulated with cetylpyridinium chloride [CPC] or chlorhexidine [CHX] prior to the DCIP assay. In comparison to placebo-treated bacterial cultures, treatment of bacterial cultures with either the CPC or CHX mouthrinse demonstrated statistically significant reductions in DCIP results by *t*-tests (*p* < 0.05).

To assess the utility of DCIP reduction assays in evaluating antimicrobial potency of commercially available mouthrinses, *A. viscosus* bacteria were incubated with serially diluted mouthwashes for 20 min. following by measurements of their reducing activity. The dose–response curves of three formulations are shown in Figure 5. Mouthwash containing cetylpiridinium chloride (CPC) exhibited the strongest effect on reducing activity of bacteria, with formulations containing chlorhexidine (CHX) also demonstrating significant effects (*p* < 0.05). In contrast, a placebo mouthwash did not affect activity even at the highest tested concentration. Likewise, when bacteria were plated on agar following incubation at 0.6% CPC, CHX and placebo mouthwashes, their counts were reduced by 5.25, 0.01 and −0.29 logs, respectively, relative to untreated control.

Treatment of *V. atypica* by both the CPC and CHX mouthwashes resulted in significantly lower DCIP mouthrinses and microbial viability in comparison to the control treatment (*p* < 0.05). Whereas the placebo mouthrinse led to no loss of microbial viability, the CPC and CHX treatments resulted in viability reductions of 1 and 5.8 logs, respectively. The corresponding results for DCIP by CPC and CHX were reductions of 12 and 19, respectively. These mouthrinses demonstrated significant effects on *V. atypica* in comparison to the placebo treatment (*p* < 0.05).

The CPC and CHX mouthrinses demonstrated significant effects on *S. mutans* bacteria in comparison to the control (*p* < 0.05). Bacterial viability following treatment by the placebo, CPC and CHX were 8.14, 4.85 and 7.46 logs, respectively, with corresponding DCIP results by the placebo, CPC and CHX treatments of 33, 4 and 15, respectively. The mouthrinses demonstrated significant treatment effects in comparison to the placebo treatment (*p* < 0.05).

[B] Toothpastes

Soluble extracts of commercially available toothpastes were prepared as described in the Materials and Methods, with amounts of extracts expressed in mg of toothpaste extracted in ml of medium. 10 mg/mL of each indicated toothpaste suspension was incubated with bacteria for 30 min. prior to the DCIP reduction assay. Treatment of *A. viscosus* with extracts of two toothpastes led to significant reductions in microbial viability (*p* < 0.05). DCIP reduction following treatment by the test and control toothpastes were 0.98 and 1.34, respectively, with corresponding reductions in microbial viability loss 4.3 and 2.7 Log CFU/mL reductions (*p* < 0.05).

*V. atypica* treated with the test and control toothpastes resulted in microbial viability reductions of 5 and 3 Log CFU/mL with corresponding DCIP reductions of 7.0 and 9.0, respectively (*p* < 0.05). Treatment of *S. mutans* by the test and control toothpastes led to reductions of 3.84 and 0.66 Log CFU/mL and corresponding DCIP reductions of 3 and 17, respectively (*p* < 0.05).

### 2.5. DCIP-Reducing Activity Following Exposure to Antibiotics

**Figure 6:**
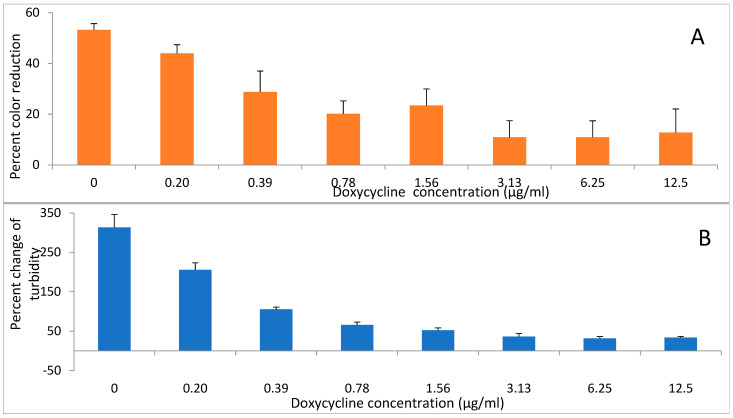
The effect of doxycycline on (**A**) DCIP-reducing activity and (**B**) growth of *A. viscosus*. Bacteria were cultured at 37 °C in anaerobic chamber for 24 h in the presence of antibiotic at the indicated concentrations. DCIP reduction assay was performed at the end of culture. The percentage change in OD at 595 nm at the end of culture relative to the start is a measure of growth. Results are shown as means of DCIP-reducing activity from triplicate samples ± standard deviation. In comparison to untreated controls, antibiotic treated bacterial cultures demonstrated statistically significant reductions in DCIP results by *t*-tests (*p* < 0.05).

**Figure 7:**
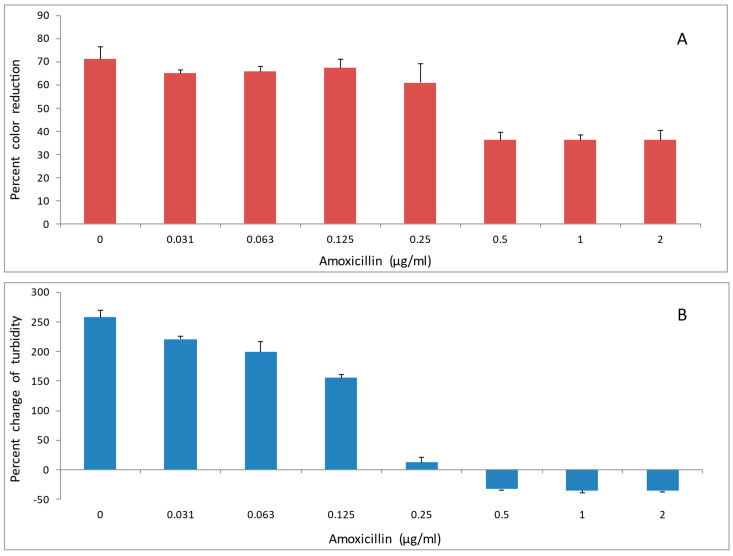
The effect of amoxicillin on (**A**): DCIP-reducing activity, and (**B**): growth, of *A. viscosus*. Bacteria were cultured at 37 °C in anaerobic chamber for 24 h in the presence of antibiotic at the indicated concentrations. DCIP reduction assay was performed at the end of culture. Percent change in OD at 595 nm at the end of culture relative to the start is a measure of growth. Results are shown as means of DCIP-reducing activity from triplicate samples ± standard deviation. In comparison to untreated controls, antibiotic treated bacterial cultures demonstrated statistically significant reductions in DCIP results by *t*-tests (*p* < 0.05).

Two antibiotics, doxycycline and amoxicillin were evaluated for their effects on reducing activity of *A. viscosus* bacteria. Incubation of bacteria with the antibiotics for up to 2 h had no detectable effect on their subsequent ability to reduce DCIP. In contrast, following 24 h growth in liquid medium in the presence of different concentrations of doxycycline (Figure 6A,B) and amoxicillin (Figure 7A,B) the activity was diminished in those samples where growth was significantly reduced or completely inhibited as determined by low turbidity relative to the untreated control.

## 3. Discussion

The redox dye DCIP has been investigated previously in a number of studies that include determining the viability of a variety of other types of bacteria [14,15], environmental organisms [18], screen for novel organisms [19] and yeasts [13,16]. Other applications report studies with eukaryotic cells [11], cultured cells such as fibroblasts [12,13] and HeLa [20], with both resting and activated neutrophils [21] demonstrating similar DCIP reduction activities [20]. DCIP reactions have also been reported from algae [22] and plants [23,24]. We were unable to find any scientific literature evaluating reductions of DCIP by oral bacteria. The present efforts evaluated the effects of oral bacteria grown in the laboratory on DCIP reduction.

A notable inclusion in the investigations were several controls incorporated in the present studies. These controls included bacteria free culture supernatants, several types of common media and evaluations of different laboratory procedures including incubation periods. Bacterial cultures did not appear to produce soluble extracellular factors for DCIP reduction with insignificant effects noted by spent bacterial culture medium. DCIP reductions were observed by viable bacterial culture that were intact. Metabolic activity of the bacterial cells influenced DCIP reductions with cell density-dependent results similar to previous reports [14,15]. Physical treatments such as heat or cold treatments decreasing the metabolic activity of bacteria led to lower DCIP responses. Corroborating previous investigations, the procedures described for DCIP reduction required no special preparation of cultures and underscores the ease of testing under standard laboratory conditions [13]. Similarly, this investigation utilized a variety of microbiological media. In the absence of bacteria, no notable background effects on DCIP reduction were observed by the evaluated microbiological media. Correspondingly, the laboratory procedures adopted included no special preparation of microbial samples, demonstrating the ease of testing with commonly available laboratory equipment using small volumes in microtiter plates [15].

Available in the scientific literature are randomized clinical studies evaluating oral health improvements after regular oral hygiene with antimicrobial mouthrinses or toothpastes [1,2,3,4]. Established antimicrobial ingredients found commonly in commercially available formulations include chlorhexidine, cetylpyridinium chloride and triclosan [3,4] with many efforts designed to evaluate ingredients with additional attributes relevant to oral hygiene or those derived from natural sources [5,6]. Commercially available formulations include a variety of additional excipients designed to provide color and flavor, along with a variety of ingredients that serve as humectants, surfactants, fluoride, gums, binding agents and fillers to provide distinct product relevant attributes. Commercially available formulations represent stable preparations compliant with regulations that are prepared under strict manufacturing processes. Commercial formulations also meet or exceed the requirements for everyday consumer use with extensive tests to optimize a variety of factors such as long-term aesthetics, product stability during transport, handling and commercial operations. The antimicrobial effects of a few established commercial formulations and a control appropriate to the evaluated formulation were evaluated on a range of oral bacteria that included both Gram-positive and Gram-negative bacteria [9,10]. In each of these instances, the test formulation demonstrated better effects than the control demonstrating the real-time efficacy outcomes relevant to prototyping to develop new formulations, optimizing formulation components the ingredients. Similarly, the procedures demonstrated the efficacy of common antibiotics used in clinical practice. Future efforts can establish guidelines for kinetic or overtime tests that offer additional insights to optimize formulations and parameters that influence activity on bacterial cells. The initial results reported in this investigation with Candida can facilitate further efforts to screen for agents appropriate for yeasts.

An additional area for investigation is the application of DCIP to elucidate microbial interactions with host cells [11,20]. This will offer a more comprehensive assessment of microbial pathogenesis that includes both prokaryotic and eukaryotic aspects or selective evaluate critical functions in these interactions. Furthermore, since DCIP has been used to examine PMN [21] and immune cells, this offers an additional avenue for future investigations.

In summary, these results describe methods that are relatively simple [15], with considerable flexibility in procedures to evaluate samples diluted serially for a variety of applications. No special handling or disposal methods are needed for results better than those of conventional approaches. These outcomes do not destroy the samples and can be conducted with common laboratory equipment. In conclusion, the procedures described can evaluate the antimicrobial effects of oral hygiene formulations and clinical samples collected from human subjects.

## 4. Materials and Methods

### 4.1. Chemicals, Buffers and Microbiological Media

2,6-Dichlorophenolindophenol sodium salt hydrate (DCIP), amoxicillin, doxycycline and antifoam 204 were obtained from Sigma-Aldrich (St. Louis, MO, USA) and stored in accordance with manufacturer’s instructions. All microbiological media were purchased from Becton Dickinson (Sparks, MD, USA). Buffers and Hanks’ Balanced Salt Solution (HBSS) were obtained from Life Technologies-Invitrogen (Carlsbad, CA, USA).

### 4.2. Bacterial Cultures

Oral bacteria *Actinomyces viscosus* (ATCC 19246), *Aggregatibacter actinomycetemcomitans* (ATCC 43718) and *Streptococcus mutans* (ATCC 25175) were cultured in Brain Heart Infusion broth, while *Veillonella atypica* (ATCC 27215) in was cultured in Peptone Yeast Extract broth, at 37 °C, in an anaerobic chamber for 48 h. Prior to experiments, bacteria were centrifuged and resuspended in fresh medium to an optical density of 0.8 (for *A. viscosus*) and 0.3 for the other strains at 600 nm.

### 4.3. DCIP Reduction Assays

To determine the relationship between viable cell counts and DCIP reduction activity, a series of dilutions were prepared, resulting in the following percentages of the original suspension: 100, 85, 70, 55, 40, 25 and 10. Aliquots of each dilution [100 μL] were placed in triplicate in wells of 96-well microplate. To each well, 100 μL of freshly prepared 80 μM solution of DCIP in medium was added. Discoloration of DCIP was measured immediately and then again after 20 min incubation with these assessments, conducted in a microplate reader (DTX 880, Beckman Coulter, Indianapolis, IN, USA) at a 600 nm wavelength. Change in DCIP color is reported as a percentage as per the following formula: (%) = 100 − ([A_600 (20 min)_/A_600 (0 min)_] × 100)^15^.

Microbial dilutions were further diluted serially and plated on Tryptic Soy agar supplemented with 5% sheep blood (Becton Dickinson, Sparks, MD, USA) using a spiral plater. All inoculated plates were incubated in an anaerobic chamber at 37 °C for atleast 48 h prior to evaluating the numbers of viable organisms.

### 4.4. The Effect of Temperature

For determination of the effect of cold or heat on DCIP-reducing activities, suspensions were incubated on ice for 60 min or in a 60 °C water bath for 45 min at a time and at temperatures indicated for each experiment. After incubations, suspensions were equilibrated at ambient temperature and their DCIP-reducing activities were measured as described above.

### 4.5. Antibiotics

Stock solutions of amoxicillin and doxycycline at concentrations of 3 mg/mL and 50 mg/mL, respectively, were prepared in sterile water. Serial dilutions of each antibiotic were prepared in microbiological medium in a microplate. Bacterial suspensions [50 µL] at an optical density of 0.5 at 600 nm were mixed with equal volumes of each serially diluted antibiotic in the same medium.

Inoculated microplates, for the evaluation of the inhibitory effects of antibiotics, were incubated for 24 h in anaerobic chamber at 37 °C. The optical densities of microplates were determined before and after incubation. DCIP was added to all plates after incubation and reduction of redox dye determined for each sample. Microbial growth inhibition was expressed as percent change in turbidity relative to the start of the culture. All assessments were conducted in triplicate.

### 4.6. Oral Care Formulations

#### 4.6.1. Toothpastes

The toothpastes evaluated in this study were commercially available and were formulated with fluoride [control, Colgate Dental Cream, New York, NY, USA] or 0.3% triclosan [test, Colgate Total]. A small amount of toothpaste was weighed and suspended at a concentration of 20 mg/mL in medium. The toothpaste slurry was briefly centrifuged to remove insoluble ingredients. Bacterial cultures were mixed with toothpaste solutions to a final concentration of 10 mg/mL, supplemented with 0.01% antifoam and incubated at room temperature for 30 min prior to the DCIP assay. Bacterial samples were also diluted and plated on blood agar to calculate viable counts.

#### 4.6.2. Mouthwashes

Commercially available mouthwashes formulated with 0.07% cetypyridinium chloride (CPC, Colgate Plax) or 0.12% chlorhexidine (CHX, Colgate Periogard) and a control formulation (placebo) without antimicrobial agents were evaluated. All tested microorganisms were grown under optimal conditions and adjusted to an optical density of 0.5 at 600 nm. Mouthwashes were serially diluted in microbiological media and 50 μL of each dilution was mixed with an equal volume of a suspension of a tested microorganism in a microplate. Controls include untreated organisms, and microbiological media with and without the evaluated treatment. DCIP was added to and incubated for 20 min at room temperature prior to evaluating DCIP-reducing activity in a spectrophotometer.

Mouthwashes were serially diluted in the medium and 50 μL of each dilution was mixed with an equal volume of a suspension of tested microorganism at OD600 of approximately 0.5. Untreated organisms and treatment-free controls were included in each experiment. After 20 min. of incubation at room temperature, DCIP-reducing activity was measured. All assessments were conducted in triplicate.

### 4.7. Statistical Analysis

Correlations between numbers of viable bacteria and DCIP reductions were examined in Microsoft Excel. The effects of physical treatments on DCIP reduction were compared to untreated controls with statistical analyses conducted by *t*-tests. The effects of oral care formulations and antibiotics on bacteria were conducted in triplicate. Controls included untreated bacteria and bacteria-free media with corresponding samples with or without the specified treatments as appropriate to complement the tests. Statistical analyses compared results from untreated controls with each treatment described and were conducted using the JMP software program.

## 5. Limitations

This study highlights the application of DCIP to evaluate the viability and effects of common oral hygiene formulations on cultures of oral microorganisms. While the present efforts detail studies with several types of oral hygiene formulations with a diversity of antimicrobial agents and formulation excipients, future investigations can evaluate other commercially available formulations or determine the effects of other agents appropriate for oral hygiene formulations. The availability of these results will progress future efforts to utilize DCIP for microbial assessments in samples collected from clinical studies.

## 6. Conclusions

Reduction of DCIP provides a simple and rapid method of quantification of live bacteria in vitro. It can be employed in the assessment of efficacy of antimicrobial oral care products, although, before being chosen as a model organism, the DCIP-reducing activities of individual bacterial strains should be evaluated in a range of concentrations of tested products.

## Figures and Tables

**Table 1:** The effects of incubating bacterial cultures at 4 °C on DCIP reduction. Shown in the table are reductions as a percentage of untreated controls.

Bacterial Strain	1 h	2 h	3 h
*A.viscosus*	80 ± 4.89	68 ± 1.33	50 ± 0.55
*V. atypica*	27 ± 1.42	23 ± 1.66	13 ± 1.83
*S. mutans*	50 ± 2.71	44 ± 1.27	37 ± 1.22
*A. actinomycetemcomitans*	55 ± 1.52	44 ± 0.89	34 ± 0.58

## Data Availability

The data supporting these findings are available upon reasonable request.
